# Long-Term Effects of a Multidisciplinary School-Based Intervention on Children’s Healthy Habits: A 1-Year Follow-Up

**DOI:** 10.3390/nu18060926

**Published:** 2026-03-15

**Authors:** Fioretta Silvestri, Davide Curzi, Giovanna Zimatore, Valerio Bonavolontà, Silvia Migliaccio, Ludovica Cardinali, Carlo Baldari, Laura Guidetti, Maria Chiara Gallotta

**Affiliations:** 1 Department of Economic, Psychological, Communication, Education and Movement Science, Niccolò Cusano University, 00166 Rome, Italy; fioretta.silvestri@unicusano.it (F.S.); davide.curzi@unicusano.it (D.C.); laura.guidetti@unicusano.it (L.G.); 2 Department of Theoretical and Applied Sciences, eCampus University, Novedrate, 22060 Como, Italy; giovanna.zimatore@uniecampus.it (G.Z.); carlo.baldari@uniecampus.it (C.B.); 3 Department of Biotechnological and Applied Clinical Sciences, University of L’Aquila, 67100 L’Aquila, Italy; valerio.bonavolonta@univaq.it; 4 Department of Experimental Medicine, Sapienza University of Rome, 00185 Rome, Italy; silvia.migliaccio@uniroma1.it; 5 Department of Life Sciences, Health, and Health Professions, Link Campus University, 00165 Rome, Italy; l.cardinali@unilink.it; 6 Department of Physiology and Pharmacology “Vittorio Erspamer”, Sapienza University of Rome, 00185 Rome, Italy

**Keywords:** nutritional intervention, physical education, childhood, obesity, eating behaviour, sedentary behaviour, school

## Abstract

**Background:** Multidisciplinary school-based interventions are considered a key strategy for promoting healthy lifestyles and preventing childhood obesity. However, evidence on the persistence of their effects beyond the intervention period remains limited. **Objectives:** This study investigated the long-term effect of different one-year combined physical education and nutritional interventions on children’s lifestyles. **Methods:** One hundred forty-five primary school children (8–10 years of age) were randomly assigned to a traditional physical education group, a coordinative physical education group, or a control group. Anthropometric variables, physical activity levels, sedentary time, and eating habits were assessed at baseline, after a 1-school-year intervention period, at 6-month follow-up, and at 1-year follow-up. An ANOVA test for repeated measures was performed to detect the among-group difference in all measured variables from baseline to 1-year follow-up over the three time points. **Results:** Physical activity levels increased significantly in both intervention groups and remained elevated at follow-up, whereas no meaningful changes were observed in the control group. Fat mass percentage increased over time in the traditional and control groups but remained stable in the coordinative group. Overall, consumption of healthy foods increased and intake of unhealthy foods decreased across time, with more pronounced improvements in children participating in physical education programs. **Conclusions:** A combined school-based nutritional and physical education intervention can produce sustained improvements in children’s lifestyle behaviours. Coordinative physical education may offer additional benefits in preventing unfavourable changes in body composition during late childhood.

## 1. Introduction

In recent years, the obesity epidemic has continued to expand worldwide, albeit at a slower pace than in the past [1]. Childhood obesity represents a significant threat to children’s health- and skill-related outcomes. Schools represent a crucial setting for promoting changes in obesity-related lifestyle factors and for inducing positive effects on children’s eating habits, physical activity levels, and sedentary behaviours [2]. The school environment offers a unique opportunity for the design, implementation, and evaluation of health interventions targeting all children, regardless of race, gender, or socioeconomic status. This is largely due to compulsory school attendance over prolonged periods and the high levels of participation and adherence typically observed in school-based interventions [3].

Recent evidence has highlighted the importance of planning integrated and multisectoral actions within school-based programs to promote healthy dietary and motor habits and to support body weight control [4]. Participation in organized sports and structured physical activity programs during the primary school years may lead to smaller increases in body mass index (BMI) during late childhood [5]. Educational research further suggests that the most effective primary strategy for improving long-term health through exercise is the development of a lifestyle characterized by regular physical activity that should be maintained into adulthood. In addition to physical activity-focused interventions, dietary literacy has been shown to influence children’s eating behaviours, thereby promoting better health outcomes among school-aged children [6]. These findings underscore the importance of identifying multidisciplinary approaches capable of increasing and sustaining children’s healthy habits over time.

Relatively few school-based preventive and therapeutic programs targeting childhood obesity have been reported, especially among preschool and primary school children; however, multicomponent interventions lasting one to two years have demonstrated significant reductions in the likelihood of children being overweight or obese, with some studies reporting decreases in the prevalence of overweight and obesity following the intervention [7]. Although short-term follow-up results (e.g., one year) appear promising, the long-term effects of such interventions (e.g., at 3, 5, or 10 years) remain largely unexplored. Encouragingly, multidisciplinary paediatric weight reduction interventions appear to be more successful than adult programs in maintaining beneficial outcomes over 5 to 10 years [8,9].

Previous studies demonstrated the effectiveness of various physical education programs combined with nutritional interventions in improving schoolchildren’s health-related behaviours [10,11,12,13], including increased physical activity levels, reduced sedentary time, and/or qualitative improvements in the consumption of selected foods [14]. However, these changes were not sufficient to elicit significant improvements in adiposity status, suggesting that school-based interventions alone may be insufficient to produce lasting anthropometric effects and highlighting the necessity of meaningful involvement of families and teachers [14]. In line with other studies reporting limited long-term outcomes of isolated school-based programs [15], these findings highlight the need for a broader, more integrated approach. Consistent evidence suggests that parental involvement plays a crucial role in establishing, monitoring, and reinforcing healthy dietary practices and daily physical activity, thereby facilitating the long-term maintenance of behaviour change [6,14]. Consequently, evaluating the persistence of behavioural changes beyond the immediate post-intervention period is essential. Follow-up assessments allow for a more accurate estimation of the real-world effectiveness of school-based interventions and their potential to influence children’s lifestyle trajectories over time.

Therefore, the purpose of the present study was to investigate children’s behaviours following a one-year intervention to assess the long-term impact of a school-based intervention on children’s lifestyles. Specifically, the aim of the present study was to evaluate the effectiveness of different physical education programs, in combination with a nutritional intervention, on schoolchildren’s healthy habits. Thus, we compared body composition, physical activity levels, sedentary time, and eating habits of primary school children at baseline (T0), after a 1-school-year intervention period (T1), at 6-month follow-up (T2), and at 1-year follow-up (T3). In this context, parental involvement is considered a critical factor in sustaining healthy behaviour over time. By addressing this gap, the present study contributes to the growing body of evidence on sustainable, multidisciplinary strategies for childhood obesity prevention.

## 2. Materials and Methods

### 2.1. Study Design and Setting

The study was designed as a cluster-randomized controlled intervention in all classes of Grade 3 and Grade 4 of three different primary schools. The unit of randomization, intervention, and analysis is the participating school [16]. The study was conducted in schools situated in a rural area approximately 50 km north of the city of Rome (Italy). To ensure comparable environmental characteristics, the study area was defined as a circle with a 5 km radius centred on a reference point. Eligibility criteria for the schools included not being already engaged in physical activity interventions and participating in the European “School Fruit Scheme” program [16]. This EU-wide voluntary scheme provided school children with fruit and vegetables, aiming to encourage good eating habits in young people [17]. Eleven classes with a total of 145 children between 8 and 10 years of age volunteered to participate in this study.

### 2.2. Participants

Eighty children in Grade 3 (8–9 years of age) and 65 children in Grade 4 (9–10 years of age) participated in the follow-up study. A total of 53 participants (23 girls and 30 boys) were in the traditional group, 53 participants (23 girls and 30 boys) were in the coordinative group, and the remaining 39 participants (16 girls and 23 boys) were in the control group.

Children were further classified into three groups based on body fat mass percentage (FM%): under fat, normal fat, or overweight/obese children, using the age- and sex-specific cut-off values proposed by McCarthy et al. [18]. Specifically, children were classified as under fat when FM% was below the 2nd percentile, normal fat when FM% ranged between the 2nd and 85th percentiles, and overweight/obese when FM% exceeded the 85th percentile. Therefore, 17 under fat, 22 normal fat, and 14 overweight/obese children were in the traditional group, 16 under fat, 22 normal fat, and 15 overweight/obese children were in the coordinative group, and 18 under fat, 16 normal fat, and 5 overweight/obese children were in the control group.

Children of the traditional and the coordinative groups participated in a combined physical education and nutritional intervention, while children of the control group participated in the European nutritional program “School Fruit Scheme” alone.

The Sapienza University Ethical Committee approved this investigation (Rif 3502 Prot. 1883/15). Written informed consent and assent was obtained from both parents and children prior to study participation.

Participants were assessed at four time points: at baseline (T0), after a 1-school-year intervention period (T1), at 6-month follow-up (T2), and at 1-year follow-up (T3).

### 2.3. Variables Assessment

#### 2.3.1. Anthropometric Variables

Anthropometric measures assessed children’s weight, height, BMI, BMI *z*-score, FM%, and lean body mass. Weight and height were measured using a scale and a stadiometer to the nearest 0.5 kg and 0.1 cm, respectively. BMI was calculated by dividing the weight (kg) by the square of the height (m). BMI *z*-score was calculated for each BMI measure with reference to age- and sex-specific limits [19,20]. Lean body mass (kg) and FM% were measured by a multi-frequency hand-to-foot bioelectrical impedance method (IOI 353 analyser; Jawon Medical Co. Ltd., Seoul, Republic of Korea).

#### 2.3.2. Physical Activity Level

Physical activity level was assessed using the Italian version of the Physical Activity Questionnaire for Older Children (PAQ-C-It) [21]. The PAQ-C is a self-administered, 7-day recall instrument designed to evaluate general levels of physical activity. It consists of nine items addressing participation in sports and games, physical activity at school, and leisure time activities, including weekends. Each item is scored on a 5-point Likert scale (1–5), and the final PAQ-C score is calculated as the mean of all item scores [22].

#### 2.3.3. Sedentary Time

Self-reported sedentary time was assessed through a parental proxy interview. Parents were asked to report the average number of minutes per week their child spent in sedentary activities outside school hours, including reading, television viewing, playing video games, and computer use [23].

#### 2.3.4. Eating Habits Measurement

Children’s eating habits were assessed using a seven-day dietary record [24]. Participants reported the usual weekly frequency of consumption of legumes, vegetables, fish, fruit, sweets, beverages, dairy products, and snacks. Response options were scored on a 7-point scale ranging from 1 (“never”) to 7 (“every day, more than once”), with intermediate categories defined as follows: 2 (“less than once a week”), 3 (“once a week”), 4 (“2–4 days a week”), 5 (“5–6 days a week”), and 6 (“once a day, every day”). The test–retest reliability coefficients for the food items ranged from 0.40 to 0.83 [24].

Prior to administration, children received instructions on how to complete the questionnaires. All questionnaires were administered in the classroom under quiet conditions, and participants were given sufficient time to complete them. A researcher was present throughout the administration to provide clarification and answer any questions from the children.

### 2.4. Intervention

#### 2.4.1. Combined Physical Education and Nutritional Intervention

The combined physical education and nutritional intervention lasted one school year.

The *physical education intervention* consisted of two 1 h sessions per week of moderate-to-vigorous physical activities.

The *traditional physical education intervention* was designed and conducted by a specialist physical education teacher with the primary goal of improving children’s endurance, strength, flexibility, and cardiovascular health, in accordance with the national curriculum guidelines [25].

The *coordinative physical education intervention*, also designed and conducted by the same specialist physical education teacher, aimed primarily to enhance children’s coordination and dexterity. This program employed a multivariate approach to physical education, emphasizing the high variability of rhythmic, gymnastic, fitness, and sport game activities to promote the multilateral development of coordinative abilities [16].

Furthermore, the specialist physical education teacher delivered brief sessions on age-appropriate physical activities, strategies to encourage daily activity, and methods for monitoring activity intensity.

The *control group* followed the traditional physical education school curriculum, delivered by the generalist classroom teacher using traditional physical education activities, in accordance with the national curriculum guidelines [25].

The *nutritional intervention* “European School Fruit Scheme” was implemented following the guidelines of the European Commission Agriculture and Rural Development: School Fruit Scheme [17]. The program aimed to increase children’s consumption of fruits and vegetables by providing fresh produce each school week and to improve their nutritional knowledge to promote healthy eating habits. The intervention covered topics such as meal planning, promoting fruit and vegetable consumption, reducing salt intake, and understanding the potential influence of mass media on dietary choices. Educational content was delivered through short lectures, games, and interactive workshops by the generalist teachers who had completed a dedicated training course [26]. Mobile applications and online resources were also provided to offer recipes and practical tips for healthier eating [27].

#### 2.4.2. Parents’ Involvement

An information campaign targeting parents was implemented through the production and distribution of educational materials [26]. The campaign aimed to reinforce and prolong the effects of school-based interventions by encouraging fruit and vegetable consumption and promoting daily physical activity, thereby supporting the maintenance of children’s healthy habits at home.

### 2.5. Statistical Analysis

An a priori power analysis to determine sample size was completed using G*Power 3.1.9.2 software [28]. A Type I alpha (error level) of 5% and a Type II beta (error level) of 5% (or a power of 95%) were set a priori. This analysis showed that for the medium effect size of 0.25 and an error probability of 0.05 and power of 0.95, the sample size would need to be N = 66. Thus, a target sample size of over 66 children was determined to account for potential attrition, thereby ensuring adequate statistical power to produce reliable and valid results [29].

Children’s baseline characteristics by intervention group (traditional group, coordinative group, control group) were described by means and standard deviations and by frequencies. Within the intervention group, differences in the baseline characteristics were verified by means of an ANOVA comparison test.

The chi-square test was then used to assess differences in weight status among the three groups throughout the intervention.

An ANOVA test for repeated measures was then performed to examine the effect of group and time on each variable to detect the among-group differences in all measured variables from baseline (T0) to 1-year follow-up over the three time points (T1, T2, T3). Significant interactions were further analysed by means of appropriate post hoc analysis. A significant group-by-time interaction indicated different time trends among the intervention groups. All results are expressed as mean ± standard deviation. Effect size was also calculated using Cohen’s definition of small, medium, and large effect size (as partial ƞ^2^ = 0.01, 0.06, 0.14) [29].

Statistical significance was defined as *p* ≤ 0.05. Statistical analysis was performed with SPSS Version 27.0 statistic software package.

## 3. Results

The children’s baseline characteristics by intervention group are shown in Table 1.

Baseline comparisons indicated that the three groups were largely comparable across anthropometric, physical activity, and dietary variables, with only a limited number of statistically significant differences and mostly small effect sizes. This suggests that class-level randomization was effective in balancing participant characteristics across intervention conditions.

Results revealed that at baseline, children of the traditional group had significantly higher BMI *z*-score and legumes consumption than children of the control group. Children of the traditional group spent a significantly higher number of minutes per week on sedentary activities than children of both the coordinative and control groups. Finally, children of the coordinative group showed a significantly lower consumption of sweets than children of both the traditional and control groups.

Globally, results revealed that the prevalence of overweight and obesity (OB) was 26.4% in the traditional group, 28.3% in the coordinative group, and 12.8% in the control group, respectively. The chi-square test detected that the proportion of OB children of the three experimental groups did not significantly change from baseline to 1-year follow-up [χ^2^ (6, n = 114) = 1.15, *p* > 0.05].

### 3.1. Anthropometric Variables

Results demonstrated that children’s body weight, body height, BMI, FM%, and lean body mass significantly increased over time (Table 2).

The time × group interaction revealed that only children of the coordinative group did not significantly change their FM% from baseline to 1-year follow-up (Table 2).

Time effects showed large effect sizes for weight, height, and lean body mass, reflecting normal growth and maturation. In contrast, time × group interaction effects were generally small, except for FM %, which showed small-to-moderate effect sizes, indicating a differential intervention-related trajectory of body composition.

### 3.2. Physical Activity Level and Sedentary Time

Our results revealed that children’s physical activity level significantly increased while sedentary time significantly decreased over time (Table 3).

The time × group interaction revealed that physical activity level of children of both the traditional and coordinative groups significantly increased while physical activity level of children of the control group remained almost unchanged from T0 to T3 (Table 3). Sedentary time of children of both the traditional and control groups significantly decreased from T0 to T1 while sedentary time of children of the coordinative group remained almost unchanged (Table 3).

Large time effects were observed for weekly physical activity level, indicating substantial and sustained increases across the study period. Time × group interaction effects were moderate to large, demonstrating that children in the intervention groups increased their physical activity to a greater extent than the control. In contrast, reductions in sedentary time were mainly explained by time effects, with only small and non-persistent time × group interactions.

### 3.3. Eating Habits

**Figure 1:**
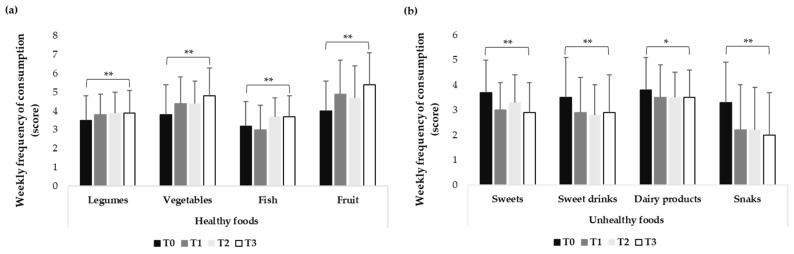
(**a**) Healthy foods changes over time (T3 vs. T0) in the whole cohort of students. (**b**) Unhealthy foods changes over time (T3 vs. T0) in the whole cohort of students. * *p* < 0.05 vs. T0. ** *p* < 0.01 vs. T0.

Children significantly changed the consumption of some specific foods over time. Consumption of healthy foods (legumes, vegetables, fish, and fruits) increased over time (Figure 1a), while consumption of unhealthy foods (sweets, sweet drinks, dairy products, and snacks) decreased over time (Figure 1b).

The time × group interaction revealed statistically significant differential effects of the group on changes of some food’s consumption over time (Table 4).

Time × group effect sizes for dietary outcomes were generally small to moderate, with larger and more persistent effects observed for fruit consumption. Reductions in unhealthy food intake, particularly sweets, showed increasing effect sizes over time, suggesting gradual but sustained behavioural changes.

## 4. Discussion

### 4.1. Anthropometric Variables

The results of our study revealed distinct physical and behavioural patterns among children participating in the traditional, coordinative, and control groups following the one-year intervention. Analysis of anthropometric variables showed that, overall, children’s body weight, height, BMI, and the percentages of fat mass and lean body mass increased significantly over time. These findings are partially in contrast with those reported by Neil-Sztramko et al. [30] who, in their review, observed that school-based physical activity interventions produced small reductions in BMI *z*-scores and had little or no effect on BMI expressed as kg/m^2^. However, important methodological differences may explain this discrepancy. While the studies included in their review implemented physical activity interventions lasting at least 12 weeks, the BMI increase observed in the present study may be attributable to its longitudinal design, which comprised a one-year intervention followed by a total observation period of approximately two years from baseline. Indeed, the observed anthropometric changes are consistent with normal growth trajectories during late childhood. Children aged 9 to 11 years typically experience marked increases in height and body weight [31], accompanied by age-related increases in BMI [31,32]. These growth-related changes should therefore be considered when interpreting intervention effects on anthropometric outcomes.

The intervention produced differential effects on FM% across the three groups. Children in both the traditional physical education group and the control group showed a significant increase in FM% at the 1-year follow-up, whereas no significant change was observed among participants in the coordinative physical education group. This finding may be explained by differences in the level of engagement and motor involvement elicited by the exercises and activities proposed in the respective interventions, with the coordinative program potentially promoting higher overall participation. The coordinative intervention was grounded in sport-based games, movement-oriented problem-solving tasks, and decision-making activities characterized by high practice variability [16]. Consistent with previous evidence, physical education programs emphasizing the development of motor competence, through the integration of locomotor, stability, and object-control skills, are associated with higher levels of adherence and sustained participation among children, particularly when activities require continuous adaptation, skill execution, and decision-making [3]. These elements likely enhanced children’s engagement, motivation, and enjoyment within the experimental group, which may have contributed to more consistent participation and greater effort invested in the proposed activities [33], and indirectly to the absence of an increase in FM% over time. Moreover, repeated exposure to varied coordinative demands is likely to support improvements in motor competence, as reflected by higher motor quotient levels [34]. Importantly, motor competence has been proposed as a key mediator between physical activity participation and health outcomes in children, with lower motor quotient levels being consistently associated with reduced physical activity engagement and a higher relative risk of children’s obesity [35]. Accordingly, the absence of an increase in FM% observed in the coordinative physical education group may be explained by a reinforcing pathway in which higher engagement leads to improvements in motor competence, which in turn supports greater participation in physical activity and more favourable body composition outcomes. Conversely, the increase in FM% observed in the traditional and control groups may reflect lower engagement and fewer opportunities to develop coordinative abilities, resulting in reduced motor competence and, ultimately, less protection against adiposity gain. While this interpretation aligns with existing theoretical models and empirical findings, the lack of direct measures of enjoyment, engagement, and motor competence represents a limitation of the present study and should be addressed in future research.

### 4.2. Physical Activity Level and Sedentary Time

Overall, students exhibited a significant increase in weekly physical activity level following the intervention, which was maintained through the second follow-up assessment. Correspondingly, sedentary time decreased after the intervention and continued to decline up to one year after its conclusion. This pattern supports the notion that combined physical activity and nutritional interventions can exert a meaningful influence on children’s physical activity levels and sedentary behaviours [36]. When considering the specific effects of the different physical education interventions, both intervention groups showed a significant increase in weekly physical activity level at the end of the intervention and at the 1-year follow-up compared with baseline values, whereas no such changes were observed in the control group. These findings are in line with previous research on school-based physical activity interventions. For instance, Kliziene et al. [37] demonstrated that an eight-month structured, school-based physical activity program significantly enhanced physical activity levels in primary school children. Similarly, earlier studies have reported that physical activity interventions conducted by specialist teachers are effective in reducing sedentary behaviour among children aged 5–12 years over time [38,39]. These results are particularly relevant given that increases in moderate-to-vigorous physical activity during childhood are a key determinant in reducing subsequent fat gain [40]. Therefore, early interventions aimed at fostering active lifestyles may have a substantial and lasting impact on children’s long-term health trajectories.

Although these findings are encouraging in school-based settings, previous research has reported that physical activity interventions implemented in schools often result in little to no increase in time spent in moderate-to-vigorous physical activity and produce minimal or no reductions in sedentary behaviour [30]. However, because the interventions included in that review ranged in duration from as little as 12 weeks, their limited effectiveness may be partly attributable to insufficient exposure time. Consequently, future research should prioritize longer-term, longitudinal interventions with extended intervention periods and follow-up assessments, as these approaches may be more effective in producing sustained changes in children’s physical activity and sedentary behaviours.

In summary, our findings suggest that physical activity-specific interventions can exert a long-term impact on children’s lifestyle behaviours. The coordinative intervention appears to have an even more pronounced effect, as indicated by the absence of an increase in FM% throughout the follow-up period. These results are consistent with previous research demonstrating the effectiveness of motor coordination-focused interventions [16], multisport interventions [41,42], and high-quality guided active play programs [43,44], which aim not only to enhance fundamental motor skills but also to prevent excess weight gain, reduce sedentary behaviour, and increase physical activity, thereby promoting children’s health [45].

### 4.3. Eating Habits

Analysis of children’s eating habits revealed significant changes in dietary behaviours over time. The entire sample showed a significant reduction in the consumption of unhealthy foods (sweets, sweet drinks, dairy products, and snacks) that was maintained up to one year after the conclusion of the intervention. Conversely, the consumption of healthy foods (legumes, vegetables, fish, and fruits) increased over the same period, with significant improvements still evident at the 1-year follow-up (Figure 1). These findings align with those of Davis et al. [46], who examined the effects of a one-year multicomponent healthy habits intervention (including a nutritional intervention) on a large cohort of primary school children. In line with our results, they reported significant increases in vegetable intake, although no reduction in BMI was observed. The observed changes are also consistent with evidence suggesting that dietary literacy and knowledge of food properties are associated with improvements in children’s health and eating behaviours [6]. Furthermore, our results confirm those of Menor-Rodriguez et al. [47], who found that primary school children (6–12 years of age) adopted healthier eating habits following a one-year nutritional education intervention.

When examining the differential effects of the physical education intervention groups, these improvements in eating habits were more pronounced among children participating in the physical education groups, suggesting that the integration of physical activity and nutritional education may have a mutually reinforcing effect in promoting healthier dietary behaviours. An increase in the consumption of healthy foods was observed among children in the physical education intervention groups, whereas a reduction in the intake of unhealthy foods was evident in both the physical education intervention groups and the control group. In contrast to our findings, Kipping et al. [48] reported no meaningful improvements in fruit and vegetable consumption among primary school children following a one-year, multicomponent intervention. However, it is important to note that, unlike the present study, their intervention comprised only 16 days of active implementation over the course of a year, which may have been insufficient to induce sustained changes in sedentary behaviour or dietary habits. In the present study, the most pronounced effects were observed in the combined physical activity and nutritional intervention groups. Notably, fruit consumption increased significantly only in the physical education groups compared with baseline values, both immediately after the intervention and at the 1-year follow-up, whereas no comparable changes were observed in the control group. Consistent with our results, previous studies reported significant increases in fruit and vegetable consumption following school-based multicomponent intervention aimed at improving physical activity and healthy eating behaviours [49,50].

Regarding unhealthy food consumption, both the control group and the traditional physical education group showed a significant decrease in the consumption of sweets and sweet drinks over time. These findings are indeed consistent with previous studies reporting long-term reductions in the consumption of packaged snacks and fruit juice [51,52], as well as high-fat foods and sugary beverages [52,53], following nutritional interventions in primary school children.

An unexpected finding was that the coordinative physical education group did not show significant changes in the consumption of unhealthy foods over time, except for a temporary reduction in snack consumption immediately after the intervention that was not maintained at follow-up. When considered alongside the previous results, this finding suggests that the combined physical activity and nutritional intervention may be more effective in sustaining the consumption of healthy foods than in consistently reducing the intake of unhealthy foods.

One possible explanation relates to the significant increase in weekly physical activity observed in the intervention groups. Higher levels of physical activity are associated with increased total energy expenditure, which may in turn lead to an increase in overall food intake [54]. Because eating habits were assessed using absolute weekly frequencies of food consumption, a general increase in energy intake may have masked relative reductions in unhealthy food consumption. This hypothesis is supported by the anthropometric findings: notably, the coordinative physical education group was the only group that did not exhibit an increase in FM% at the 1-year follow-up. This pattern suggests that improvements in dietary quality, reflected by increased intake of healthy foods, combined with increases in physical activity, may have contributed to more favourable body composition outcomes.

Overall, the findings of the present study are consistent with previous research reporting that multidisciplinary school-based interventions integrating physical activity and nutritional components can improve body composition [55] and contribute to the long-term prevention of overweight and obesity in children [11,56].

### 4.4. Strength and Limitations

A key strength of the present study lies in its longitudinal design. Interventions conducted during childhood and aimed at modifying lifestyle behaviours require extended observation periods to determine whether improvements in healthy habits are sustained over time. Therefore, the longitudinal approach adopted in this study allowed for the evaluation of both immediate and longer-term effects of the intervention. Nevertheless, as many lifestyle behaviours are resistant to change within relatively short timeframes, future research should further investigate the optimal duration of interventions necessary to produce lasting behavioural changes. An additional strength of this study is the inclusion of different types of physical education interventions. Beyond physical activity per se, the qualitative characteristics of the activities implemented may play a critical role in influencing children’s engagement and adherence during physical activity sessions [3], potentially exerting indirect effects on the development of children’s long-term lifestyle habits.

Overall, the intervention produced large and sustained effects on physical activity levels, small-to-moderate intervention-specific effects on fat mass trajectory, and modest but meaningful changes in dietary behaviours, particularly fruit intake and sweet consumption. Effect sizes were consistent with expectations for school-based, real-world interventions and indicate behavioural changes of potential public health relevance without interference with children’s normal growth.

This study has some limitations. First, since the sample was recruited from schools participating in the “School Fruit Scheme” and, for ethical reasons, all students were required to take part in the program, it was not possible to include a control group that did not receive any intervention (neither nutritional nor physical activity-related). Second, although anthropometric outcomes were assessed, the inclusion of measures of motor competence would have provided additional insight into whether the type of physical education intervention differentially influenced motor skill development, a key factor during childhood growth. Third, previous research suggests that physical activity interventions may be more effective in primary school girls than in boys [57]; therefore, sex-specific analyses could have offered further understanding of intervention effects. However, such analyses would have required a larger sample size than the sample available in the present study.

Finally, parental role is an interpretive hypothesis rather than an empirically tested variable. Future research should use questionnaires administered to parents to examine parental involvement more closely, as this may represent a crucial component for enhancing the effectiveness and sustainability of school-based health promotion programs [14].

## 5. Conclusions

Taken together, the findings of the present study suggest that interdisciplinary educational interventions conducted by specialist physical education teachers are effective in enhancing children’s knowledge of diet, nutrition, and active lifestyles, thus promoting healthier behaviours over the long term [2].

From this perspective, high-quality physical education may serve as a foundation for lifelong engagement in physical activity and sport. The learning experiences provided through physical education lessons can support the development of psychomotor skills, cognitive understanding, as well as social and emotional competencies that are essential for maintaining an active and healthy lifestyle. Despite the strong evidence supporting the role of physical education in child development, only approximately 60% of Italian primary schools offer at least two hours of physical education per week during curricular time (and these lessons are frequently delivered by generalist teachers without specific expertise in physical education teaching) [58]. For this reason, the present study strongly supports the implementation of professionally guided physical education interventions that enable children to participate in well-structured physical activity programs led by specialist physical education teachers.

Finally, the results of the present study may have important public health implications, as longitudinal evidence indicates that even modest increases in moderate-to-vigorous physical activity during childhood are associated with a substantially lower risk of developing obesity later in life [59]. Extending the investigation of lifestyle behaviours across educational stages represents a key future direction. Preliminary evidence from university students suggests that the promotion of healthy lifestyles remains relevant beyond childhood and adolescence [60].

However, to improve consistency and comparability across studies in this field, it remains crucial to continue implementing well-structured, targeted nutritional and physical activity interventions in primary school settings, with the aim of optimizing long-term health outcomes for children.

## Figures and Tables

**Table 1:** Baseline characteristics of subjects by intervention groups (N = 145).

Variable	Traditional Group	Coordinative Group	Control Group	*p*	Partial ƞ^2^
	(n = 53)	(n = 53)	(n = 39)		
Weight (kg)	35.6 ± 9.5	36.8 ± 8.7	33.5 ± 11.3	0.287	0.018
Height (cm)	134.4 ± 7.4	136.2 ± 6.6	134.6 ± 7.1	0.378	0.014
BMI (kg/m^2^)	19.6 ± 3.7	19.8 ± 4.0	18.3 ± 4.7	0.168	0.025
BMI *z*-score	0.9 ± 1.1	0.7 ± 1.2	0.2 ± 1.1 *	0.014	0.064
FM (%)	19.1 ± 8.3	18.8 ± 9.5	15.5 ± 8.7	0.136	0.029
Lean body mass (kg)	28.8 ± 5.6	29.3 ± 4.8	27.9 ± 6.8	0.510	0.010
Weekly physical activity level (score)	2.1 ± 0.7	2.3 ± 0.6	2.1 ± 0.8	0.296	0.017
Sedentary time (min/week)	691.6 ± 230.6	428.0 ± 220.5 *	519.5 ± 210.1 *	<0.001	0.213
Legumes (score)	3.8 ± 1.5	3.5 ± 1.1	3.1 ± 1.2 *	0.038	0.045
Vegetables (score)	3.9 ± 1.5	4.0 ± 1.5	3.3 ± 1.8	0.075	0.036
Fish (score)	3.2 ± 1.3	3.2 ± 1.2	3.0 ± 1.3	0.705	0.005
Fruit (score)	3.7 ± 1.4	4.2 ± 1.7	4.4 ± 1.7	0.141	0.027
Sweet (score)	4.1 ± 1.6	3.0 ± 1.4 * ^§^	4.1 ± 1.7	<0.001	0.104
Sweet drink (score)	3.3 ± 1.8	3.3 ± 1.6	4.0 ± 1.7	0.066	0.038
Dairy product (score)	3.9 ± 1.7	4.0 ± 1.6	3.8 ± 1.6	0.812	0.003
Snack (score)	3.5 ± 1.8	2.8 ± 1.6	3.5 ± 1.4	0.303	0.047

* *p* < 0.05 vs. traditional group; ^§^ *p* < 0.05 vs. control group. Weekly physical activity level score ranges 1 (none)–5 (very often). Usual weekly frequency of food consumption score ranges 1 (never)–7 (every day, more than once).

**Table 2:** Changes in anthropometric data after intervention and through 1-year follow-up by the intervention group.

Variable	Time	Intervention Groups Pooled							*p* Value—Change from Baseline	Partial ƞ^2^
Weight (kg)	T0				35.7 ± 10.0					
	T1				36.5 ± 10.3				<0.001	0.365
	T2				40.4 ± 11.2				<0.001	0.725
	T3				41.1 ± 11.4				<0.001	0.766
Height (cm)	T0				134.8 ± 7.0					
	T1				135.6 ± 7.0				<0.001	0.192
	T2				140.0 ± 7.4				<0.001	0.799
	T3				141.7 ± 7.3				<0.001	0.875
BMI (kg/m^2^)	T0				19.4 ± 4.2					
	T1				19.6 ± 4.3				0.002	0.076
	T2				20.4 ± 4.4				<0.001	0.385
	T3				20.2 ± 4.3				<0.001	0.331
BMI *z*-score	T0				0.7 ± 1.1					
	T1				0.7 ± 1.1				0.875	0.000
	T2				0.7 ± 1.1				0.134	0.021
	T3				0.7 ± 1.1				0.554	0.003
FM (%)	T0				18.6 ± 8.5					
	T1				19.3 ± 8.2				<0.001	0.148
	T2				20.7 ± 8.2				<0.001	0.420
	T3				19.8 ± 8.2				<0.001	0.197
Lean body mass (kg)	T0				28.9 ± 5.7					
	T1				29.3 ± 5.9				<0.001	0.216
	T2				32.2 ± 6.0				<0.001	0.861
	T3				33.1 ± 6.2				<0.001	0.871
**Variable**	**Time**	**Traditional Group**			**Coordinative Group**	**Control Group**			*p* Value * Group by Time	**Partial** **ƞ^2^**
Weight (kg)	T0	35.8 ± 9.5			36.9 ± 8.8	33.9 ± 12.0				
	T1	36.5 ± 9.7			37.7 ± 9.1	34.5 ± 12.5			0.206	0.026
	T2	41.3 ± 11.0			41.0 ± 9.7	38.4 ± 13.3			0.081	0.040
	T3	42.0 ± 11.5			41.9 ± 10.0	38.9 ± 12.9			0.122	0.034
Height (cm)	T0	134.4 ± 7.0			135.9 ± 6.8	133.9 ± 7.2				
	T1	135.5 ± 7.1 *			136.2 ± 6.8	135.0 ± 7.4 *			0.034	0.054
	T2	140.5 ± 7.2 *			140.77 ± 7.4 *	138.5 ± 7.4 *			0.010	0.073
	T3	142.0 ± 7.2 *			142.5 ± 7.4 *	140.1 ± 7.2 *			0.039	0.052
BMI (kg/m^2^)	T0	19.6 ± 3.8			19.9 ± 4.0	18.6 ± 5.0				
	T1	19.6 ± 3.8			20.2 ± 4.0	18.9 ± 5.1			0.061	0.045
	T2	20.7 ± 4.0			20.6 ± 4.0	19.7 ± 5.4			0.165	0.029
	T3	20.5 ± 4.1			20.5 ± 4.1	19.5 ± 5.0			0.259	0.022
BMI *z*-score	T0	0.9 ± 1.1			0.9 ± 1.1	0.2 ± 1.1				
	T1	0.8 ± 1.2			0.9 ± 1.0	0.3 ± 1.1			0.310	0.021
	T2	0.9 ± 1.1			0.9 ± 1.0	0.4 ± 1.1			0.539	0.011
	T3	0.8 ± 1.2			0.8 ± 1.0	0.3 ± 1.1			0.312	0.021
FM (%)	T0	19.4 ± 7.8			19.4 ± 9.2	16.3 ± 8.3				
	T1	19.6 ± 7.7			20.2 ± 9	17.3 ± 7.6			0.100	0.044
	T2	21.5 ± 7.7 *			20.7 ± 8.9 *	19.4 ± 7.8 *			0.031	0.065
	T3	20.6 ± 7.7 *			19.9 ± 9.1	18.6 ± 7.6 *			0.040	0.061
Lean body mass (kg)	T0	28.4 ± 5.5			29.5 ± 4.7	28.5 ± 7.4				
	T1	28.7 ± 5.7			29.9 ± 4.9	29.3 ± 7.6			0.098	0.049
	T2	32.1 ± 6.1			32.7 ± 4.9	31.7 ± 7.5			0.179	0.036
	T3	33.1 ± 6.3			33.5 ± 5.2	32.5 ± 7.6			0.091	0.050

* *p* < 0.05 vs. baseline.

**Table 3:** Changes in weekly physical activity level and sedentary time after intervention and through 1-year follow-up by intervention group.

Variable	Time	Intervention Groups Pooled							*p* Value—Change from Baseline	Partial ƞ^2^
Weekly physical activity level (score)	T0				2.2 ± 0.7					
	T1				2.6 ± 0.8				<0.001	0.428
	T2				2.7 ± 0.9				<0.001	0.323
	T3				2.9 ± 0.9				<0.001	0.371
Sedentary time (min/week)	T0				552.4 ± 248.9					
	T1				496.3 ± 216.6				0.002	0.068
	T2				469.3 ± 229.3				<0.001	0.123
	T3				444.3 ± 238.5				<0.001	0.158
**Variable**	**Time**	**Traditional Group**			**Coordinative Group**	**Control Group**			*p* Value * Group by Time	**Partial** **ƞ^2^**
Weekly physical activity level (score)	T0	2.1 ± 0.7			2.3 ± 0.6	2.1 ± 0.8				
	T1	2.7 ± 0.8 *			2.9 ± 0.7 *	2.2 ± 0.9			<0.001	0.177
	T2	3 ± 0.9 *			2.9 ± 0.9 *	2.3 ± 0.8 *			<0.001	0.106
	T3	3.1 ± 0.9 *			3.2 ± 0.9 *	2.3 ± 0.7			<0.001	0.109
Sedentary time (min/week)	T0	694.7 ± 231.7			430.0 ± 222.0	519.5 ± 210.1				
	T1	618.4 ± 202.1 *			435.8 ± 182.5	411.0 ± 205.8 *			0.041	0.045
	T2	607.1 ± 199.9			360.2 ± 202.2	425.5 ± 209.2			0.866	0.002
	T3	571.6 ± 234.6			323.4 ± 180.1	429.6 ± 228.4			0.817	0.003

* *p* < 0.001 vs. T0. Weekly physical activity level score ranges 1 (none)–5 (very often).

**Table 4:** Changes in eating habits after intervention and through 1-year follow-up by intervention group.

Healthy Foods	Time	Traditional Group	Coordinative Group	Control Group	*p* Value * Group by Time	Partial ƞ^2^
Legumes (score)	T0	3.8 ± 1.5	3.5 ± 1.1	3.1 ± 1.2		
	T1	3.5 ± 0.9	3.8 ± 1.1 *	4.2 ± 1.2 *	<0.001	0.124
	T2	4.2 ± 1.1	3.7 ± 1.2	3.8 ± 1.0	0.759	0.005
	T3	4.1 ± 1.2	3.8 ± 1.2	4.0 ± 1.2	0.265	0.025
Vegetables (score)	T0	3.9 ± 1.5	4.0 ± 1.5	3.3 ± 1.8		
	T1	4.5 ± 1.3	4.7 ± 1.3	3.8 ± 1.6	0.870	0.003
	T2	4.8 ± 1.2	4.0 ± 1.3	4.1 ± 1.0	0.110	0.041
	T3	5.3 ± 1.4	4.7 ± 1.6	4.1 ± 0.9	0.446	0.015
Fish (score)	T0	3.2 ± 1.3	3.2 ± 1.2	3.0 ± 1.3		
	T1	2.6 ± 1.1 *	3.2 ± 1.4	3.5 ± 1.2	0.016	0.075
	T2	3.6 ± 0.8	3.9 ± 1.0	3.7 ± 1.0	0.446	0.015
	T3	3.6 ± 0.9	4.2 ± 1.1	3.3 ± 1.3	0.084	0.046
Fruit (score)	T0	3.7 ± 1.4	4.2 ± 1.7	4.4 ± 1.7		
	T1	5.2 ± 1.6 *	5.3 ± 1.9 *	3.9 ± 1.4	0.002	0.111
	T2	5.5 ± 1.7 *	4.1 ± 1.5	4.3 ± 1.5	<0.001	0.145
	T3	6.0 ± 1.3 *	5.0 ± 1.8 *	5.0 ± 1.8	0.007	0.089
**Unhealthy Foods**	**Time**	**Traditional Group**	**Coordinative Group**	**Control Group**	*p* Value * Group by Time	**Partial** **ƞ^2^**
Sweets (score)	T0	4.1 ± 1.6	3.0 ± 1.4	4.1 ± 1.7		
	T1	3.1 ± 1.7	2.1 ± 1.4	4.2 ± 1.3	0.329	0.021
	T2	3.3 ± 2.1 *	3.2 ± 1.3	3.7 ± 1.3 *	0.048	0.056
	T3	2.5 ± 1.3 *	3.1 ± 0.9	3.2 ± 1.2 *	<0.001	0.167
Sweet drinks (score)	T0	3.3 ± 1.8	3.3 ± 1.6	4.0 ± 1.7		
	T1	2.8 ± 1.6	2.6 ± 1.6	3.7 ± 1.6	0.948	0.001
	T2	2.9 ± 1.1	2.7 ± 0.8	2.6 ± 1.4 *	0.039	0.060
	T3	3.0 ± 1.1	2.9 ± 1.1	2.8 ± 1.0 *	0.044	0.057
Dairy products (score)	T0	3.9 ± 1.7	4.0 ± 1.6	3.8 ± 1.6		
	T1	3.4 ± 1.7	3.4 ± 1.5	3.5 ± 1.5	0.946	0.001
	T2	4.0 ± 1.5	3.4 ± 0.8	3.0 ± 0.9	0.072	0.048
	T3	3.6 ± 1.4	3.4 ± 0.8	3.3 ± 1.2	0.528	0.012
Snacks (score)	T0	3.5 ± 1.8	2.8 ± 1.6	3.5 ± 1.4		
	T1	1.8 ± 0.8 *	2.0 ± 1.2 *	3.4 ± 1.4	0.005	0.095
	T2	2.0 ± 0.8	2.2 ± 0.9	2.7 ± 1.5	0.148	0.035
	T3	1.9 ± 0.8	2.1 ± 0.9	1.9 ± 0.7	0.069	0.049

* *p* < 0.05 vs. baseline. Usual weekly frequency of food consumption score ranges 1 (never)–7 (every day, more than once).

## Data Availability

The data presented in this study are available on request from the corresponding author (M.C.G.) due to privacy restrictions.

## Abbreviations

The following abbreviations are used in this manuscript:

BMI	Body mass index
FM%	Body fat mass percentage
PAQ-C	Physical Activity Questionnaire for Older Children
